# Generation of Therapeutically Potent Spheroids from Human Endometrial Mesenchymal Stem/Stromal Cells

**DOI:** 10.3390/jpm11060466

**Published:** 2021-05-25

**Authors:** Alisa Domnina, Larisa Alekseenko, Irina Kozhukharova, Olga Lyublinskaya, Mariia Shorokhova, Valeriy Zenin, Irina Fridlyanskaya, Nikolay Nikolsky

**Affiliations:** Department of Intracellular Signaling and Transport, Institute of Cytology, Russian Academy of Sciences, Tikhoretsky pr. 4, 194064 St. Petersburg, Russia; al.l.l@mail.ru (L.A.); kojuxarova@mail.ru (I.K.); o.lyublinskaya@mail.ru (O.L.); shili-mariya@yandex.ru (M.S.); naturfox@yandex.ru (V.Z.); irfreed@yahoo.com (I.F.); vitaamid@mail.ru (N.N.)

**Keywords:** endometrial mesenchymal stem cells, cell spheroids, serum-free conditions, wound healing

## Abstract

Endometrial mesenchymal stem/stromal cells (eMSCs) hold great promise in bioengineering and regenerative medicine due to their high expansion potential, unique immunosuppressive properties and multilineage differentiation capacity. Usually, eMSCs are maintained and applied as a monolayer culture. Recently, using animal models with endometrial and skin defects, we showed that formation of multicellular aggregates known as spheroids from eMSCs enhances their tissue repair capabilities. In this work, we refined a method of spheroid formation, which makes it possible to obtain well-formed aggregates with a narrow size distribution both at early eMSC passages and after prolonged cultivation. The use of serum-free media allows this method to be used for the production of spheroids for clinical purposes. Wound healing experiments on animals confirmed the high therapeutic potency of the produced eMSC spheroids in comparison to the monolayer eMSC culture.

## 1. Introduction

Mesenchymal stem/stromal cells (MSCs) are multipotent clonogenic cells of mesodermal origin [1,2]. MSCs’ differentiation potential, ease of harvest, low immunogenicity, capacity to promote cell migration and angiogenesis make them an attractive therapeutic remedy [3,4]. MSCs can be isolated from numerous adult tissues and then expanded in vitro due to their strong adherence to cell culture plastic. Initially, MSCs were isolated from bone marrow but have since been obtained from many other adult tissues, including the endometrium (eMSCs) [5,6,7,8,9,10]. eMSCs have been identified as CD146+/CD140b+ clonogenic cells of perivascular origin which can be isolated either from an endometrial biopsy or even from menstrual blood [11]. eMSCs have shown great potential for various regenerative medicine approaches [10,11,12]. It is considered that they promote the survival and proliferation of host cells through paracrine activity that enhances angiogenesis as well as reducing inflammation [11,12]. It was shown that eMSCs exhibited increased overall function when aggregated into three-dimensional (3D) spheroids [13,14]. eMSC spheroids secrete more vascular endothelial growth factor (VEGF), prostaglandin E2 (PGE2) and other key factors involved in tissue regeneration [13,14]. For eMSC expansion, animal-derived materials are usually applied. Most commonly, fetal bovine serum (FBS) is used. The presence of FBS in eMSC cultures limits both clinical and research applications of the cells, compromises the safety of clinical application and presents economic and ethical restrictions [15,16,17,18,19,20]. To solve these issues, different alternatives to FBS-supplemented cell culture media are being developed, such as xeno-free and serum-free systems [21,22,23,24,25,26]. The latter, among other things, ensure the standardization of cell culturing conditions, which is crucial for the development of eMSC-based therapy approaches. However, to produce therapeutically potent eMSC spheroids, the procedure of spheroid formation needs to be standardized as well. Here, we developed an approach for the formation of standardized eMSC spheroids suitable for therapeutic application and examined a therapeutic property of these 3D eMSC spheroids cultured in serum-free media for wound healing in a rat model.

## 2. Materials and Methods

### 2.1. Cells

In our experiments, we used eMSCs, MSCs isolated from adipose tissue (adMSCs) and MSCs isolated from bone marrow (bmMSCs) obtained from the MSC collection of the Department of Intracellular Signaling of the Institute of Cytology RAS, Russia. eMSCs were established from endometrium fragments of menstrual blood taken from five healthy women at the age of 25–35 years, as it was previously described [10]. Briefly, endometrium blood samples were collected and centrifuged with PBS. Precipitates were plated in 6-cm Petri dishes (Corning, Corning, NY, USA) in standard cultural media containing DMEM/F12 medium with 10% fetal bovine serum (FBS) (Life Technologies, Carlsbad, CA, USA), 1% antibiotic–antimycotic mixture and 1% Glutamax or in serum-free media described below and cultivated for 3–7 days. The media were changed every 3rd day. After the cell culture reached confluence, the cells were subcultured with EDTA/trypsin solution. eMSCs at passages 3–5 were used to study their properties and at passages 7, 10 and 22 to determine the effectiveness of spheroid formation on different passages.

### 2.2. Serum-Free Media

In this study, we tested two different media: serum-free medium 1 (SFM 1) StemPro MSC SFM (Life Technologies, Carlsbad, CA, USA) (used according to the producer’s protocol), and serum-free medium 2 (SFM 2), composed according to the following protocol [21]. It contains DMEM/F12, 0.25% recombinant human albumin (Sigma-Aldrich, St. Louis, MO, USA), 1% chemically defined lipid concentrate (CDLC) (Life Technologies, Carlsbad, CA, USA), 1 x insulin-transferrin-selenium (Life Technologies, Carlsbad, CA, USA), 10 ng/mL platelet-derived growth factor-AB (Sigma-Aldrich, St. Louis, MO, USA), 10 ng/mL basic fibroblast growth factor (Life Technologies, Carlsbad, CA, USA), 5 ng/mL transforming growth factor-b1 (Sigma-Aldrich, St. Louis, MO, USA), 10 ng/mL epidermal growth factor (Sigma-Aldrich, St. Louis, MO, USA), 1% Glutamax (Sigma-Aldrich, St. Louis, MO, USA), 1% antibiotic–antimycotic mixture and 0.1% fibronectin from human plasma (Sigma-Aldrich, St. Louis, MO, USA) for the cell dish covering. The cells were subcultured with CTS TrypLE Select Enzyme (Life Technologies, Carlsbad, CA, USA).

### 2.3. Spheroid Formation

All eMSC lines used for spheroid generation were tested for surface markers expression. Cells were negative for CD34, CD45 and HLA-DR, and positive for CD44, CD90, CD73, CD105, CD146 and CD140b (Figure S2). Spheroids were formed from eMSCs, adMSCs or bmMSCs using the hanging drop technique. A total of 7000 cells per 35 μL were placed in drops on the cover of 10-cm Petri dishes (Corning, Corning, NY, USA) and inverted. Cells spontaneously aggregated in hanging drops for 48 h. After that, eMSC spheroids (totally 1 × 10^6^ cells) from the same serum-free media group were transferred into 6-cm Petri dish coated with 2-hydroxyethylmethacrylate (HEMA, Sigma-Aldrich, St. Louis, MO, USA), for the ease of further collection for cell analysis and/or transplantation. The culture medium was completely changed to the fresh one. The viability of eMSCs in spheroids was monitored using flow cytometry. For obtaining the single-cell suspension, spheroids from the dish coated with 2-hydroxyethylmethacrylate, together with the culture medium, were transferred to a 15 mL conical tube and centrifuged for 3 min at 1500 rpm. After centrifugation, the supernatant was removed; 10 mL of PBS solution was added, and the tube was centrifuged again. Then, the PBS solution was removed, and spheroids were incubated with 0.05% trypsin/EDTA solution for 5 min at +37 °C. After the incubation was complete, the cell suspension was gently pipetted 2–3 times to achieve spheroid dissociation. Finally, a three-fold volume of the culture media was added for trypsin inactivation. To assess eMSC viability, cells were stained with propidium iodide (50 μg/mL) and, after that, analyzed with the flow cytometer CytoFLEX (Beckman Coulter, Brea, CA, USA).

### 2.4. Selection of Conditions for Spheroid Formation

To define the appropriate conditions for the production of uniform-sized spheroids, we added to the standard culture medium different concentrations of chemically defined lipid concentrate CDLC (0.01%, 0.05% or 1%) (Life Technologies, Carlsbad, CA, USA) and formed eMSC spheroids by the hanging drop method, as described above. Spheroid areas were measured using ImageJ software. No less than 50 spheroids were analyzed in each case. To evaluate the size heterogeneity, the area was normalized to the mean spheroid area in the sample.

### 2.5. Immunophenotyping

Immunophenotyping of monolayer eMSCs and eMSCs from spheroids was performed with an Epics XL flow cytometer (Beckman Coulter, Brea, CA, USA). The single-cell suspension from spheroids was obtained as described above in Section 2.3 and resuspended in PBS with 5% FBS at a concentration of 1 × 10^6^ cells/mL. The monolayer culture in the flask was washed with PBS solution. Then, 0.05% trypsin/EDTA solution was added, and cells were incubated for 5 min at + 37 °C; after that, a three-fold volume of culture media was added, and the cell number was counted. Then, the cell suspension was centrifuged for 3 min at 1500 rpm, resuspended in PBS with 5% FBS at a concentration of 1 × 10^6^ cells/mL and used for further immunophenotyping. FITC- and PE (phycoerythrin)-conjugated antibodies to CD34, CD 44, CD45, CD90, CD 146 and HLA-1 and to CD73, CD105 and HLA DR were applied. All antibodies, including the isotype control antibodies, were purchased from Beckman Coulter (Indianapolis, IN, USA), except for CD140b (#323606), which was purchased from BioLegend (San Diego, CA, USA). Incubation with antibodies was performed according to the manufacturer’s instructions. Typically, antibodies (3 μL) were added to 30,000 cells and were incubated at 4 °C for 45 min in the dark. The final IgG concentration varied in the range of 0.5–1 µg/mL depending on the CD lot used.

### 2.6. Adipogenic Differentiation

Here, 2 × 10^4^ cells/cm^2^ were seeded in Petri dishes coated with 0.1% gelatin (Sigma-Aldrich, St. Louis, MO, USA). When the cells reached about 80% confluence, 1 mM dexamethasone (Sigma-Aldrich, St. Louis, MO, USA), 0.5 mM isobutyl-methyl-xanthine (IBMX; Sigma-Aldrich, St. Louis, MO, USA), 10 μg/mL human recombinant insulin (Sigma-Aldrich, St. Louis, MO, USA) and 100 mM indomethacin were added to the culture medium. The cells were differentiated for 3–5 weeks with a half volume of the medium changed every 2–3 days. Lipid drops were visualized with Oil Red staining (Sigma-Aldrich, St. Louis, MO, USA) according to the manufacturer’s instructions.

### 2.7. Osteogenic Differentiation

Here, 2 × 10^4^ cells/cm^2^ were seeded in Petri dishes coated with 0.1% gelatin. After the cells reached 100% confluence, 100 nM dexamethasone, 10 mM β glycerol phosphate and 0.2 mM ascorbate- 2- phosphate were added to the culture medium. The cells were differentiated for 3–5 weeks with a half volume of the medium changed every 2–3 days. Then, the cells were fixed with 70% cold ethanol for 1 h and stained with Alizarin Red, pH 4.1 (Sigma-Aldrich, St. Louis, MO, USA).

### 2.8. qRT-PCR Assays

To analyze gene expression, total RNA was isolated with RNesy Micro Kit (Qiagen, Hilden, Germany) according to the manufacturer’s instructions. RNA was quantified in the NanoDrop ND-1000 Spectrophotometer (Thermo Fisher Scientific, Waltham, MA, USA). cDNA was obtained by reverse transcription of RNA using the RevertAid H Minus First Strand cDNA Synthesis Kit (Fermentas, Waltham, MA, USA) according to the manufacturer’s instructions. It was then amplified with specific primers, using DreamTaq™ PCR Master Mix (2X) (Thermo Fisher Scientific, Waltham, MA, USA) with CycloTempamplificator. qRT-PCR cDNA was amplified with specific primers, using EvaGreen^®^ dye (Thermo Fisher Scientific, Waltham, MA, USA) and DreamTaq™ PCR Master Mix (2X) (Thermo Fisher Scientific, Waltham, MA, USA) in the BioRad CFX-96 real-time system (BioRad, Hercules, CA, USA) according to the protocol. Expression of target genes was normalized to the GAPDH gene. Primers and reaction conditions are presented in Table S1 [27,28,29,30]. All amplifications were performed in triplicates. Experiments were repeated at least three times.

### 2.9. Animals

All experiments were performed with male Wistar rats, weighing 230–250 g. The animals were maintained in the designated animal care facility with free access to tap water and food. All experimental procedures with animals were performed according to the institutional guidelines for the care and use of laboratory animals. All studies on animals were performed after approval by the Institutional Animal Care and Use Committee of the Institute of Cytology RAS (Assurance Identification number F18-00380).

### 2.10. Animal Wound Model

Eighteen adult albino male Wistar rats with 230–250 g weight were used in experiments. Animals were divided into 3 groups. Group 1—transplantation of eMSCs cultured on standard culture medium (*n* = 6); group 2—transplantation of eMSCs cultured on SFM1 (*n* = 6); group 3—on SFM2 (*n* = 6). All manipulations were conducted under aseptic conditions. Animals were anesthetized by intramuscular injection of Zoletil 100 (Virbac, Carros, France) at a dose of 5 mg/kg weight. The animal backs were shaved and disinfected with 70% alcohol. Three pieces of full-thickness skin (1.5 × 1.5 cm) on the central side of the back were excised to create three full-thickness skin wounds. Immediately after suspension of eMSCs cultured in monolayer (4 × 10^6^ cells/per wound) or eMSCs in spheroids (4 × 10^6^ cells/per wound), they were injected in 100 µL PBS solution around the prepared full-thickness skin wounds. The same volume of PBS without cells was used on the control wound. The territories of the wounds were then photographed. Finally, each animal was covered with a bandage.

### 2.11. Wound Healing Assay

On the day of surgery and every day after the open wounds were photographed, the wound area was measured by tracing the wound margin and calculating the pixel area using ImageJ. The time of wound closure was defined as the time at which the wound bed was completely re-epithelialized. The percentage of wound closure was calculated as follows: (area of original wound minus area of the actual wound)/area of original wound × 100% [31]. A wound was considered completely closed when the wound area was equal to zero. Some rats were sacrificed 7 days after surgery for histological examination.

### 2.12. Histology

Collected skin tissue was fixed in 10% buffered formalin for 24 h and embedded in paraffin. The tissue blocks were cut into 5-mm-thickness sections followed by routine hematoxylin and eosin staining. Epithelialization (length of the regenerating epithelia) and granulation tissue thickness were assessed by light microscopy. No less than 5 histological sections were measured in each case. 

### 2.13. Statistical Analysis

The data are presented as the mean ± standard deviation (SD) when indicated. The differences in recovery of granulation and epithelium tissues in rat wounds between PBS and eMSCs or PBS and spheroids were tested by the Kruskal–Wallis H-test (non-parametric ANOVA) followed by post hoc pairwise comparison using Dunn’s test. All other data were treated using Student’s *t*-test (two-tailed). Data were analyzed with GraphPadPrism software (GraphPad software, San Diego, CA, USA). Differences were considered significant at *p* < 0.05.

### 2.14. Ethical Approval

This study was approved by the Institutional Animal Care and Use Committee of the Institute of Cytology RAS (Assurance Identification number F18-00380).

### 2.15. Statement of Human and Animal Rights

All experimental procedures involving animals in this study were conducted following the institutional guidelines for the care and use of laboratory animals and approved by the Institutional Animal Care and Use Committee of the Institute of Cytology RAS (Assurance Identification numberF18-00380).

## 3. Results

### 3.1. Production of Uniform-Sized Spheroids

**Figure 1:**
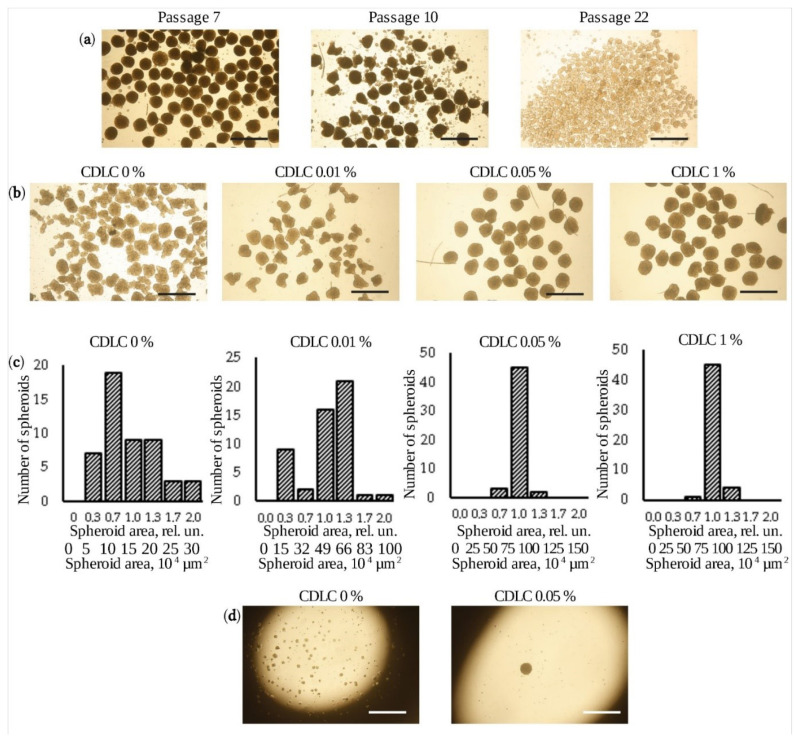
Generation of uniform-sized eMSC spheroids. (**a**) Aggregation of eMSCs into spheroids on different passages (scale bar, 600 μm); (**b**) formation of uniform-sized eMSC spheroids in serum-containing medium supplemented with different concentrations of CDLC (scale bar, 600 μm); (**c**) distribution of eMSC spheroid areas obtained from the analysis of the bright-field images of spheres generated in serum-containing medium supplemented with different concentrations of CDLC; the upper labels display the area normalized to the mean value in the sample, and the lower labels display the area in absolute numbers. (**d**) Phase-contrast microscopy showing the aggregation of eMSCs into a spheroid in a hanging drop in serum-containing medium with and without 0.05% CDLC after 48 h (scale bar, 600 μm).

We have previously shown that using hanging drops is a practical method to form spheroids from eMSCs [13,14]. However, in this work, while studying the possibility of increasing the yield of spheroids and testing cell lines obtained from different donors, we faced the problem of obtaining spheroids that varied in size. We found that, sometimes, not all cells in one droplet aggregate together, but several smaller spheroids can be formed. Furthermore, it was also observed that the ability of eMSCs to form spheroids depended on the donor and dropped with the cell culture passage (Figure 1a). This circumstance makes it necessary to select the cell lines most suitable for the formation of spheroids, which prevents the use of autologous cells in the clinical use of spheroids. To overcome the emerging problems, we set a goal to modify the composition of the culture medium applied in the process of spheroid formation in order to increase their yield and quality. We found that the addition of chemically defined lipid concentrate (CDLC) to the culture medium to create spheroids promoted the aggregation of all cells in the droplet and resulted in the formation of uniformly sized spheroids. CDLC is a lipid emulsion added to serum-free media for the cultivation of various cell lines. We analyzed the size of the forming spheroids using different concentrations of CDLC. It was estimated that the addition of 0.01% CDLC to the growth medium increased the yield and average size of spheroids but did not reduce their size variability. The 0.05% CDLC resulted in enhanced yield, spheroid enlargement and decreased size variability (Figure 1b,c). A further increase in the concentration of CDLC to 1% did not exert an additional effect. The efficiency of 0.05% CDLC was tested on five eMSC lines from different donors, including donors with a reduced ability to form spheroids, as well as on eMSCs at various passages (passage 5, passage 10, passage 22). In all cases, the use of the culture medium modified by CDLC reduced the size variability of forming spheroids (Figure S1c). It was also found that when CDLC was added, only one spheroid was formed from all cells in the droplet (Figure 1d). A similar effect of the CDLC-modified medium on spheroid formation was registered for MSCs obtained from bone marrow and adipose tissue (Figure S1a,b).

### 3.2. Formation of Spheroids Using Serum-Free Media

**Figure 2:**
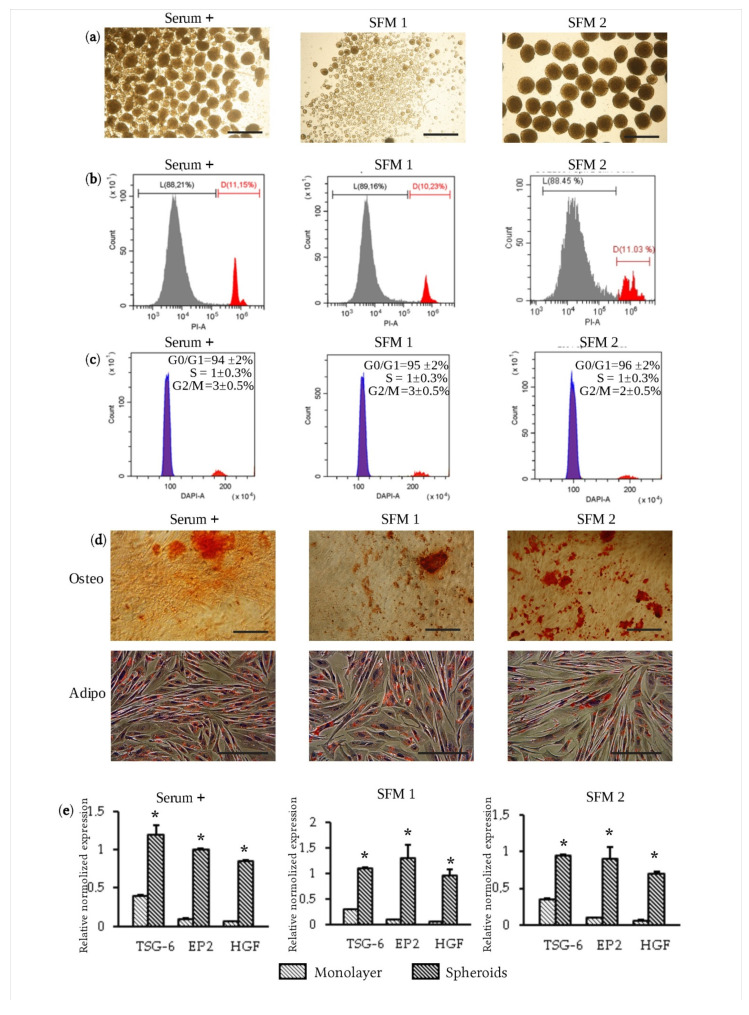
Viability, multipotency, cell cycle distribution and gene expression of eMSC spheroids under serum-free conditions. (**a**) Aggregation of eMSCs into spheroids cultured in serum-containing medium (Serum+), SFM 1 or SFM 2 (scale bar, 300 μm.); (**b**) viability of eMSCs in spheroids cultured in serum-containing medium (Serum+), SFM 1 or SFM 2; L—live cells, D—dead cells; (**c**) representative cell cycle distribution of eMSCs in spheroids cultured in serum-containing medium (Serum+), SFM 1 or SFM 2. (**d**) Osteogenic and adipogenic differentiation of eMSCs in spheroids cultured in serum-containing medium (Serum+), SFM 1 or SFM 2 (scale bar, 200 μm). (**e**) q-RT-PCR assay of TSG6, EP2 and HSF genes in eMSC spheroids and monolayer cells under serum-free conditions. Data are shown as mean ± SD (*n* = 3). Two-tailed Student’s t-test was utilized for pairwise comparison. * *p* < 0.01 vs. eMSCs in monolayer.

Another problem of eMSC application for clinical purposes is the use of media containing components of animal origin for their cultivation. The main component of the medium traditionally utilized for the cultivation of MSCs is the animal serum. Today, various culture media containing substances that replace serum are being actively developed. To determine the possibility of using such media for the formation of spheroids from eMSCs, we used two serum-free media of different compositions and the medium with serum as a control. SFM 1 is manufactured commercially, whilst SFM 2 is composed in accordance with the protocol proposed earlier [21] and contains the addition of 1% CDLC. As expected, when using the SFM 1 medium that did not contain CDLC, we observed the formation of small spheroids that were not uniform in size (Figure 2a). The use of the SFM 2 medium led to the formation of uniform-sized spheroids, and their number corresponded to the number of droplets (Figure 2a). Interestingly, modification of the SFM 1 medium with the addition of 0.05% CDLC significantly improved spheroid formation (Figure S1d). We evaluated the properties of eMSC spheroids formed in the standard culture medium and in SFM 1 and SFM 2 media in terms of viability, ability to differentiate into osteoblasts, adipocytes, expression of CD markers and therapeutically significant genes. eMSCs dissociated from spheroids after 3 days of their cultivation retained a high level of viability regardless of the composition of the culture medium (Figure 2b). eMSCs from spheroids formed in serum-free media did not differ in their ability to differentiate into osteoblasts and adipocytes from eMSCs cultured on the standard medium with serum (Figure 2d). It is known that MSCs in spheroids stop proliferation and accumulate in the G0/G1 phase of the cell cycle [13,14,32]. In eMSCs, when cultivated in spheroids, G0/G1 block was also observed, regardless of the composition of the medium (Figure 2c). The evaluation of CD marker expression revealed a declined expression of CD146, common for eMSCs in spheroids (Table 1) [13,14,32]. However, we found that if eMSCs were dissociated from spheroids and cultured as 2D cells, expression of CD 146 was restored in 72 h (Figure S2). The expression of other markers in eMSCs organized in spheroids retained the pattern typical for MSCs (Table 1). eMSCs cultured in spheroids displayed the higher expression of the main therapeutic genes, such as the anti-inflammatory TNF alpha-induced protein 6 (TSG-6), the hepatocyte growth factor (HGF) and stimulating angiogenesis factor (prostaglandin EP2 receptor), compared with eMSCs cultured in monolayer, regardless of the composition of the culture medium (Figure 2e). Thus, we have shown that eMSCs compacted into spheroids in the serum-free medium do not significantly alter their properties characteristic of eMSCs cultivated in monolayer and supposedly exhibit an enhanced therapeutic potential.

### 3.3. Therapeutic Efficacy of eMSC Spheroids in Serum-Free Medium

**Figure 3:**
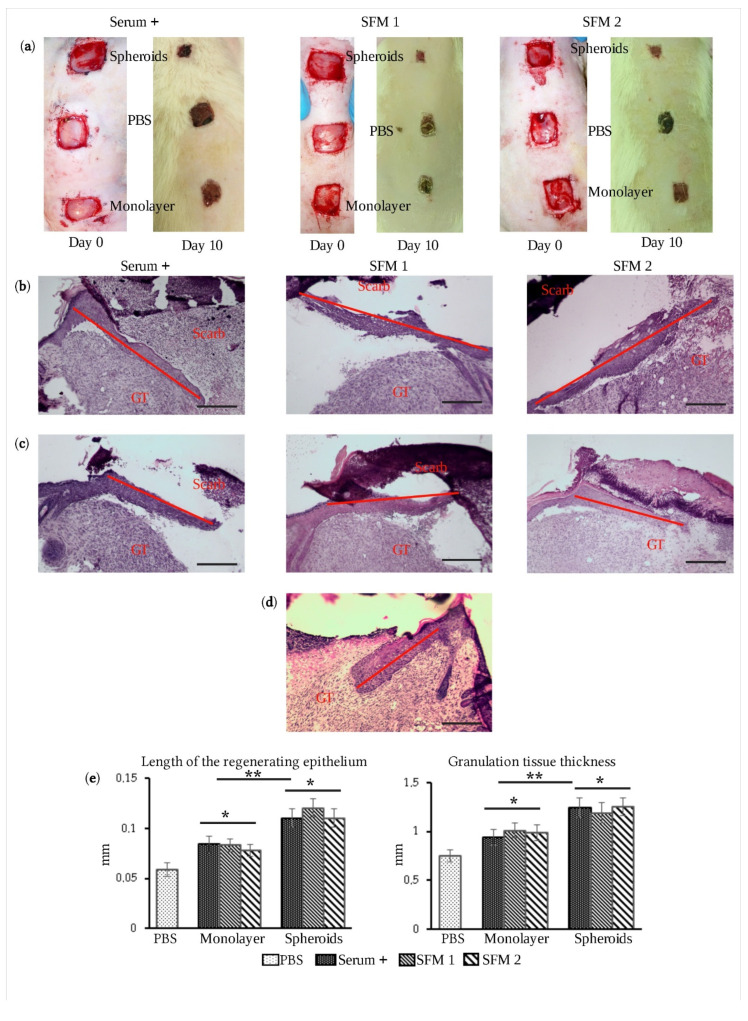
Therapeutic efficacy of eMSC spheroids in serum-free medium. (**a**) Macroscopic view of the wound closure in rat after transplantation of eMSCs in spheroids cultured in serum-containing medium (Serum+), SFM 1 or SFM 2, eMSCs cultured in monolayer at the same conditions and PBS as a control; (**b**) H&E staining of paraffin-embedded tissue section of rat wounds on the 7th day after transplantation of eMSCs in spheroids (scale bar, 100 μm). GT—granulation tissue, line—regenerating epithelium. (**c**) H&E staining of paraffin-embedded tissue section of rat wounds on the 7th day after transplantation of eMSCs cultured in monolayer (scale bar, 100 μm). GT—granulation tissue, line—regenerating epithelium. (**d**) H&E staining of paraffin-embedded tissue section of rat wounds on the 7th day after PBS injection (scale bar, 100 μm). GT—granulation tissue, line—regenerating epithelium. (**e**) Recovery of granulation and epithelium tissues in rat wound after eMSC transplantation. Data are shown as mean ± SD (*n* = 15). The differences in recovery of granulation and epithelium tissues in rat wounds were tested by the Kruskal–Wallis H-test (non-parametric ANOVA) followed by post hoc pairwise comparison using Dunn’s test. * *p* < 0.05 vs. PBS control; ** *p* < 0.05 vs. monolayer culture at the same conditions.

We have previously demonstrated that transplantation of eMSCs in spheroids improved wound healing in rats [14]. To assess the therapeutic potential of eMSCs in spheroids organized in a serum-free medium, we also used a wound healing model. To form wounds in rats, skin flaps on the back were removed. The transplantation of eMSC spheroids formed in the serum-free medium was performed by their injections along the edge of the wound. The effect of transplantation was compared to one produced by the spheroids formed in the FBS-supplemented medium. In each spheroid group, PBS solution and eMSCs cultured in monolayer at the same conditions were used as controls. We observed that transplantation of eMSCs in spheroids accelerated wound healing (Figure 3a). On day 10, in animals transplanted with eMSC spheroids cultured in both serum-free media, the wound area was almost completely covered with a new epithelium compared with the wound closure after transplantation of cells cultured in monolayer at the same conditions or injection of PBS solution (Figure S3b). The average time of wound closure for eMSC transplantation in spheroids was 12 days compared with the administration of PBS solution (15 days) (Figure S3a). Examination of histological sections revealed an increased length of the newly formed regenerating epithelium in wounds after transplantation of eMSC spheroids, compared with monolayer eMSC transplantation or injection of PBS solution (Figure 3b–d). Measurements of the thickness of granulation tissue also showed improved regeneration after transplantation of eMSCs in spheroids (Figure 3e). In conclusion, we have shown that transplantation of eMSC spheroids generated in serum-free media stimulated wound healing in rats more efficiently than transplantation of eMSCs grown as a monolayer culture. The method we applied for the generation of uniform-sized spheroids in media that do not contain components of animal origin looks promising for clinical use.

## 4. Discussion

Cell therapy based on MSC transplantation is applied in clinics as a new treatment strategy for different diseases [1]. Numerous clinical trials in which MSCs are used for the cure of various tissue and organ disorders are in progress (ClinicalTrials.gov; search for trials containing the term “Mesenchymal Stem Cells”). Recent studies showed that MSCs can be obtained from the endometrial tissue [8,9,10,11]. The human endometrium is a dynamic tissue that every month undergoes periods of regeneration, differentiation and desquamation. During the menstrual cycle, 4–10 mm of endometrial tissue is shed. eMSCs are located around blood vessels in both the functionalis and basalis endometrial layers and are shed into menstrual fluid as the functionalis breaks down during menstruation [11]. A desquamated endometrium in menstrual blood has been proven to be the most available and noninvasive source of human MSCs [10,11]. These cells meet the criteria of the International Society for Cell Therapy for MSCs and exhibit multipotency, high proliferation activity during long-term cultivation, genetic stability and low immunogenicity that make them a promising source of stem cells for clinical applications [2,8,9,10,11,33]. It is assumed that the therapeutic MSC effect is mediated via the secretion of trophic factors, such as angiogenic and pro- and anti-inflammatory molecules [3,4,11,12]. The latest reports demonstrated that the aggregation of MSCs into 3D multicellular spheroids enhanced their paracrine secretion, cell survival after transplantation, homing, stemness, differentiation potential and angiogenic and anti-inflammatory properties [34,35,36,37,38]. It is considered that aggregation in spheroids closely repeats the in vivo MSC niche by providing spatial cell organization with increased cell–cell interactions [34,35,36,37,38]. eMSCs in spheroids exhibit all multipotent properties of eMSCs in monolayer, such as differentiation potential and expression of key CD markers, but have higher levels of angiogenic and anti-inflammatory gene expression [13,14]. eMSCs cultured in spheroids have been successfully applied for the restoration of the rat endometrium in an Asherman’s syndrome model, as well as for skin wound healing in rats [13,14].

For cell isolation and cultivation in vitro, culture media containing FBS are usually used. However, for broad clinical applications of MSCs, this approach has many drawbacks [15,16,17,18,19]. FBS is generally ill defined, and immunologic reactions against xenogeneic antigens cannot be excluded. It has been shown that FBS proteins associate with major histocompatibility complex (MHC) class I, leading to T cell proliferation after cell transplantation even in an autologous setting [24]. Pathogens such as mycoplasma, viruses, prions and endotoxins cannot be excluded completely as well [16]. Lastly, there is an ethical point of view because FBS is harvested from bovine fetuses. As a result, different suitable alternatives are being developed and tested [21,22,23,24,25,26]. Although human MSC spheroids are regarded as a highly promising cell source for regenerative medicine, only a few studies have been devoted to the possibility of their maintenance under serum-free conditions [39,40]. Our current investigation shows that eMSCs organized in spheroids retain their stem cell properties and ability to differentiate into mesodermal lineages under serum-free conditions. Despite the decreased expression of CD 146 (melanoma cell adhesion molecule, MCAM) in eMSC spheroids, this marker is restored after eMSCs return back to monolayer culture. Earlier, we demonstrated that eMSCs aggregated into spheroids undergo cytoskeleton reorganization that leads to a cell size reduction and a round cell morphology [13,14]. There is an opinion that the enzymatic dissociation of spheroids to a single-cell suspension can disrupt sensitive phenotypic molecules [38], but we rather suggest that cells in spheroids may have different, in comparison to monolayer cultures, cell–cell interactions and therefore distinct expression patterns of cell adhesive molecules including CD 146.

In this study, we found that the effectiveness of eMSC spheroid formation depends on various factors, such as the donor, culture passage and the properties of the medium. Previously, it was shown that MSCs cannot condense into tight spheroids when cultured in several commercial stem cell media, and only supplementation with human serum albumin (HSA) can result in compact MSC spheroid formation [40]. We found that, instead of HSA, the addition of 0.05% chemically defined lipid concentrate (CDLC) to the culture medium can be used for spheroid formation. CDLC is a concentrated lipid emulsion that contains non-animal-derived fatty acids (arachidonic, linoleic, linolenic, myristic, oleic, palmitic, stearic) designed to reduce or partially replace fetal bovine serum in cell culture media. We show that CDLC supplementation leads to the formation of spheroids with controllable sizes and a uniform distribution. At the same time, all the properties of eMSCs in spheroids formed in the CDLC-supplemented serum-free medium are preserved, including a more than ten times increased expression of the main therapeutic genes (TSG-6; EP2; HGF) and the ability to stimulate tissue regeneration. Current data show that transplantation of eMSCs in spheroids under serum-free conditions reduces the average time for complete wound closure and enhances re-epithelialization in rats. Our findings on the high therapeutic potential of eMSC spheroids under serum-free conditions in an animal model provide a promising basis for their further clinical use. Moreover, the proposed method of spheroid formation can also be applied to various tissue engineering techniques. For example, generation of uniform-sized spheroids is extremely important to produce functional tissues from modular tissue blocks (building blocks) represented by spheroids using bioprinting and bioassembly approaches [41].

## 5. Conclusions

Human endometrial stem/stromal cells (eMSCs) in monolayer or organized in spheroids maintained in serum-free conditions sustain their basic properties. eMSC spheroids generated in serum-free media applied for wound healing in a rat model were more therapeutically effective than the cells in monolayer. We suggest that an improved therapeutic effect is implemented via enhanced secretion of angiogenic and anti-inflammatory factors. This study proposes using serum-free conditions for eMSC spheroid formation and application. Modification of the media with CDLC improves spheroid formation. The addition of 0.05% CDLC to the culture medium leads to the formation of spheroids with controllable sizes and a uniform size distribution. Overall, our findings highlight the possibility to maintain eMSC spheroids under serum-free conditions in CDLC-supplemented media as a promising tool for future clinical research and therapy.

## Figures and Tables

**Table 1:** CD expression in eMSC monolayer and spheroids under different culture conditions.

CD Marker	eMSC	Spheroids	eMSC SFM1	Spheroids SFM1	eMSC SFM2	Spheroids SFM2
CD34	0.18 ± 0.3%	0.32 ± 0.1%	0.60 ± 0.2%	0.39 ± 0.1%	0.45 ± 0.3%	0.15 ± 0.1%
CD 44	100.00 ± 2%	93.14 ± 2%	99.57 ± 2%	92.35 ± 2%	98.48 ± 2%	96.48 ± 2%
CD 45	0.83 ± 0.5%	0.76 ± 0.2%	0.94 ± 0.2%	0.66 ± 0.2%	0.93 ± 0.2%	0.74 ± 0.1%
CD 73	99.62 ± 1%	94.36 ± 2%	99.86 ± 2%	96.68 ± 2%	93.95 ± 3%	95.95 ± 2%
CD 90	95.66 ± 2%	96.34 ± 2%	96.66 ± 2%	99.34 ± 2%	93.07 ± 2%	99.96 ± 1%
CD 105	92.90 ± 2%	94.89 ± 2%	98.39 ± 1%	91.89 ± 2%	93.92 ± 2%	90.20 ± 2%
CD 146	97.95 ± 2%	3.09 ± 1%	99.95 ± 1%	0.56 ± 0.3%	95.00 ± 2%	0.50 ± 0.1%
HLA-1	99.59 ± 2%	99.99 ± 2%	99.59 ± 2%	98.44 ± 2%	98.17 ± 2%	97.20 ± 2%
HLA-DR	0.31 ± 0.1%	0.30 ± 0.2%	0.60 ± 0.2%	0.38 ± 0.1%	0.50 ± 0.2%	0.60 ± 0.1%

## Data Availability

The datasets used and/or analyzed during the current study are available from the corresponding author on reasonable request.

## Supplementary Materials

The following are available online at https://www.mdpi.com/article/10.3390/jpm11060466/s1, Table S1: Primer sequences for control and target genes and Q-PCR conditions, Figure S1: Production of uniform-size spheroids from MSCs of different origin. (a) Aggregation of MSCs from adipose tissue into spheroids (passage 5) (scale bar, 600 μm); (b) aggregation of MSCs from bone marrow into spheroids (passage 5) (scale bar, 600 μm); (c) aggregation of eMSCs after prolonged cultivation into spheroids (passage 22) (scale bar, 600 μm); (d) aggregation of eMSCs cultured in SFM 1 into spheroids (scale bar, 600 μm), Figure S2: Expression of CD146 and CD 140b in eMSCs. (a) High expression of CD 146 in monolayer culture; (b) high expression of CD 140b in monolayer culture; (c) reduced expression of CD 146 in eMSCs dissociated from spheroids; (d) restoration of CD 146 expression in eMSCs dissociated from spheroids and cultured in monolayer for 72 h, Figure S3: Wound healing dynamic in rat model. (a) Average time of wound closure after eMSC transplantation (average for all culture conditions). Data are shown as mean ± SD (*n* = 9). Two-tailed Student’s t-test was utilized for pairwise comparison. * *p* < 0.05 vs. PBS control; ** *p* < 0.05 vs. monolayer culture. (b) Wound area covered with new epithelium on day 10 in rats after transplantation. Data are shown as mean ± SD (*n* = 9). The differences in wound closure were tested by the Kruskal–Wallis H-test (non-parametric ANOVA) followed by post hoc pair-wise comparison using Dunn’s test. * *p* < 0.05 vs. PBS control; ** *p* < 0.05 vs. monolayer culture at the same conditions.
