# Supplementary material for: Generation of Therapeutically Potent Spheroids from Human Endometrial Mesenchymal Stem/Stromal Cells
Source: J Pers Med. 2021 May 25;11(6):466. doi: 10.3390/jpm11060466 (PMC8229788; doi:10.3390/jpm11060466)
Supplement: Supplementary file 1 [file jpm-11-00466-s001.zip › jpm-1187398-supplementary.pdf]

Supplementary Materials:

Table S1. Primer sequences for control and target genes and Q-PCR conditions.

Symbol	Primer sequence	Amplification conditions	PCR productsize (bp)	Accession number	Reference
TSG-6	S: GATGGATGGCTAAGGGCAGAGT-3′<br>AS: TCATTTGGGAAGCCTGGAGATT-3′	93°C, 20 s, 61°C, 20 s72°C 30 s	208	NM_007115.3	[38]
EP2	S: 5-CCACCTCATTCTCCTGGCTA-3<br>AS: 5-CGACAACAGAGGACTGAACG-3	93°C, 20 s, 62°C, 20 s72°C 30 s	216	NM_000956.3	[39]
HGF	S: 5′-CTCACACCCGCTGGGAGTAC-3′<br>AS: 5′-TCCTTGACCTTGGATGCATTC-3′	93°C, 20 s, 62°C, 20 s72°C 30 s	104	XM_011516115.2	[40]
GAPDH	S: 5′-GACTCATGACCACAGTCCATGC-3′<br>AS: 5′-AGAGGCAGGGATGATGTTCTG-3′	93°C, 20 s, 67°C, 20 s72°C 30 s	112	NM_001289746.1	[41]

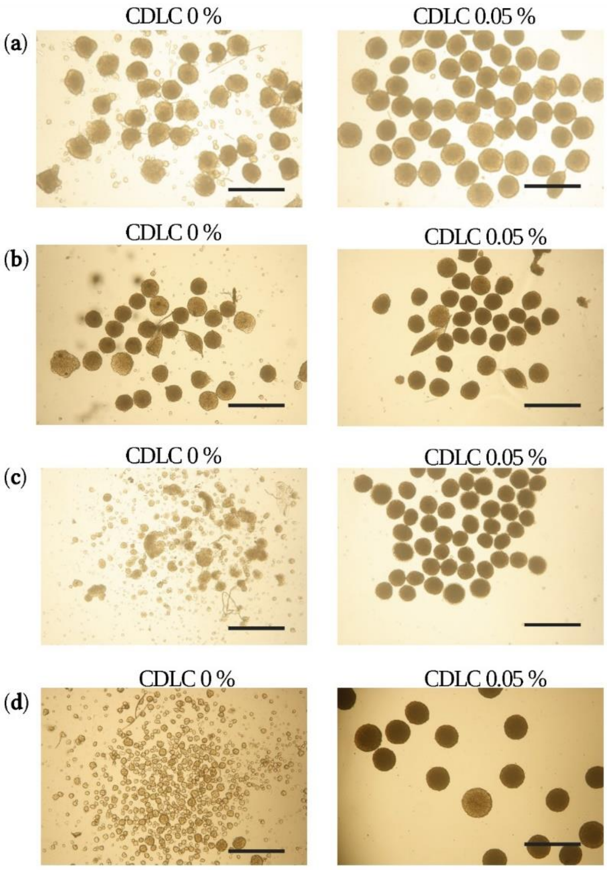

Figure S1. Production of uniform-size spheroids from MSC of different origin. (**a**) Aggregation of MSC from adipose tissue into spheroids (passage 5) (Scale bar, 600 μm.); (b)Aggregation of MSC from bone marrow into spheroids (passage 5) (Scale bar, 600 μm.); (**c**) Aggregation of eMSC after prolonged cultivation into spheroids (passage 22) (Scale bar, 600 μm.); (**d**) Aggregation of eMSC cultured in SFM 1 into spheroids (Scale bar, 600 μm.).

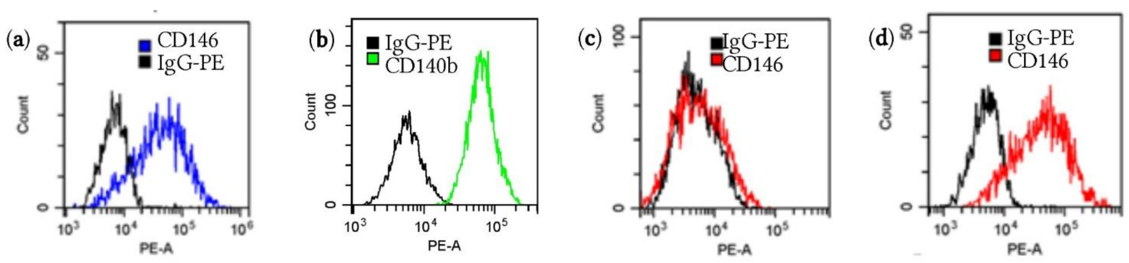

Figure S2. Expression of CD146 and CD 140b in eMSC. (a) High expression of CD 146 in monolayer culture; (b) High expression of CD 140b in monolayer culture; (c) Reduced expression of CD 146 in eMSC dissociated from spheroids; (d) Restoration of CD 146 expression in eMSC dissociated from spheroid and cultured in monolayer for 72 h.

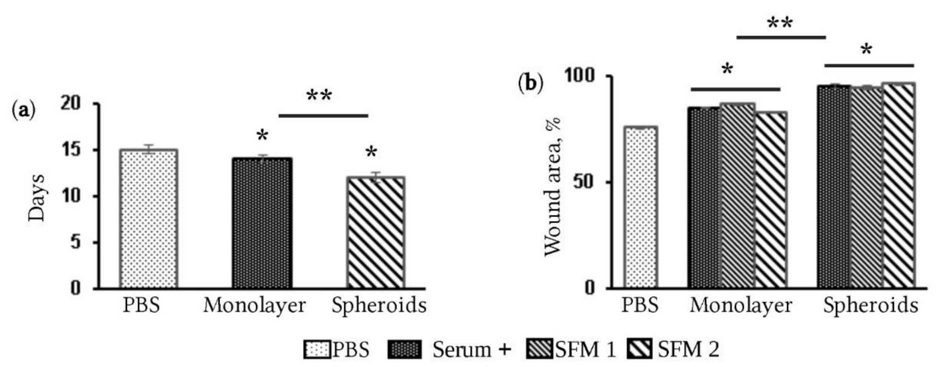

Figure S3. Wound healing dynamic in rat model. (a) Average time of wound closure after eMSC transplantation (average for all culture conditions). Data are shown as mean ± SD, (n=9). Two-tailed Students t test was utilized for pairwise comparison. *p<0.05 vs. PBS control; **p<0.05 vs. monolayer cultured. (b)Wound area covered with new epithelium on day 10 in rats after transplantation . Data are shown as mean ± SD, (n=9). The differences in wound closure were tested by Kruskal-Wallis H-test (non-parametric ANOVA) followed by post hoc pair-wise comparison using Dunn's test.. *p<0.05 vs. PBS control, **p<0.05 vs. monolayer cultured at the same conditions..
